# High, clustered, nucleotide diversity in the genome of *Anopheles gambiae *revealed through pooled-template sequencing: implications for high-throughput genotyping protocols

**DOI:** 10.1186/1471-2164-10-320

**Published:** 2009-07-16

**Authors:** Craig S Wilding, David Weetman, Keith Steen, Martin J Donnelly

**Affiliations:** 1 Vector Group, Liverpool School of Tropical Medicine, Pembroke Place, Liverpool, L3 5QA, UK

## Abstract

**Background:**

Association mapping approaches are dependent upon discovery and validation of single nucleotide polymorphisms (SNPs). To further association studies in *Anopheles gambiae *we conducted a major resequencing programme, primarily targeting regions within or close to candidate genes for insecticide resistance.

**Results:**

Using two pools of mosquito template DNA we sequenced over 300 kbp across 660 distinct amplicons of the *An. gambiae *genome. Comparison of SNPs identified from pooled templates with those from individual sequences revealed a very low false positive rate. False negative rates were much higher and mostly resulted from SNPs with a low minor allele frequency. Pooled-template sequencing also provided good estimates of SNP allele frequencies. Allele frequency estimation success, along with false positive and negative call rates, improved significantly when using a qualitative measure of SNP call quality. We identified a total of 7062 polymorphic features comprising 6995 SNPs and 67 indels, with, on average, a SNP every 34 bp; a high rate of polymorphism that is comparable to other studies of mosquitoes. SNPs were significantly more frequent in members of the cytochrome p450 mono-oxygenases and carboxy/cholinesterase gene-families than in glutathione-S-transferases, other detoxification genes, and control genomic regions. Polymorphic sites showed a significantly clustered distribution, but the degree of SNP clustering (independent of SNP frequency) did not vary among gene families, suggesting that clustering of polymorphisms is a general property of the *An. gambiae *genome.

**Conclusion:**

The high frequency and clustering of SNPs has important ramifications for the design of high-throughput genotyping assays based on allele specific primer extension or probe hybridisation. We illustrate these issues in the context of the design of Illumina GoldenGate assays.

## Background

Mapping of loci controlling traits of interest in the malaria vector mosquito *Anopheles gambiae *is dependent upon the availability of suitable genomic markers. Quantitative trait locus (QTL) mapping analyses in *An. gambiae *have successfully employed polymorphic microsatellites [1] – the utility of which can be readily predicted and verified – to study insecticide resistance [2,3], *Plasmodium *refractoriness and encapsulation [4-6] or hybrid sterility [7,8]. However, microsatellites occur too infrequently in most genomes to permit fine scale mapping. By contrast, single nucleotide polymorphisms (SNPs) are usually abundant; but extensive discovery and validation work is required before their application. This has represented a major obstacle to the development of association mapping approaches in *An. gambiae*.

The release of the complete genome sequence of the PEST strain of *An. gambiae *in 2002 [9] provided significant information on polymorphism, with nearly 450,000 SNPs reported. However, the PEST strain is a cross between two molecular forms (considered incipient species in *An. gambiae*): a long-term M-form laboratory strain originating from Nigeria and field-collected S-forms from Western Kenya, crossed with additional Kenyan S-forms. As such, the SNPs identified in the PEST sequence are expected to be biased towards those that segregate between the M and S molecular forms, rather than SNPs likely to be polymorphic within and among natural populations. In addition, SNPs are at relatively low frequency in the PEST genome (approximately 1 segregating site every 620 bp) and have an uneven distribution across the genome, resulting in a paucity of SNPs in many chromosomal divisions (Fig. 3 in [9]). To date, published resequencing studies in *An. gambiae *have validated some of the PEST genome SNPs, uncovered additional SNPs, and provided additional information on polymorphism levels, but have been of small scale and/or focussed primarily on genes involved in immunity [10-12].

We are interested in the factors controlling resistance to insecticides in *An. gambiae*. Gene expression studies using the *An. gambiae *Detox-chip [13] – a microarray for the study of genes putatively involved in insecticide metabolism – have identified loci overexpressed in insecticide resistant strains [14-16]. However, gene expression studies are unable to detect resistance arising from allelic variants, or to locate the regulatory elements underpinning gene expression. Association mapping has the power to detect such variants and therefore represents a powerful complementary approach. In its current form (version 3) the *An. gambiae *Detox chip [13] has probes for 254 genes including cytochrome p450 monooxygenases, glutathione-S-transferases and carboxy/cholinesterases, plus members of other gene families potentially involved in detoxification processes (peroxidases, reductases, superoxide dismutases, ATP-binding cassettes), and housekeeping loci which serve as controls.

The primary aim of our study was to resequence the suite of genes present on the Detox chip microarray to provide data for development of a highly multiplexed SNP array for association mapping of insecticide resistance in *An. gambiae*. Our resequencing used pooled genomic DNA (gDNA) as template, and we also evaluate the performance of the pooling technique with respect to accuracy in allele frequency detection and Type I and II error rates for SNP discovery. SNPs to be screened in highly multiplexed approaches, such as the Illumina GoldenGate assay [17] and Affymetrix Genechip assay [18], must not only be identified, validated and exhibit suitable levels of polymorphism, but must also be flanked by sequences free of additional polymorphisms that may interfere with the assay. Therefore, the other major aim of our study was to gain insight into the distribution of SNPs in the *An. gambiae *genome, and how this impacts the design of highly-multiplexed arrays. Information on all SNPs discovered in the present study are freely available in public access databases.

## Methods

### Samples

In order to incorporate high diversity and reduce sequencing time and costs, two pools of gDNAs were created from *An. gambiae *M- and S-forms of diverse geographical origin. The M pool consisted of samples from Odumasy, coastal Ghana (*N *= 3), Bonia, northern Ghana (*N *= 3) and Koubri, southern Burkina Faso (*N *= 4) and the S pool consisted of samples from Odumasy, Ghana (*N *= 3), Mampong, central Ghana (*N *= 2), Asembo Bay, Kenya (*N *= 2) and Thyolo, Malawi (*N *= 3). DNA from each sample was extracted using the Ballinger-Crabtree method [19] and molecular form (M/S) determined with the method of Fanello *et al*. [20]. The 2La^+^/2La inversion kayotype was determined using the PCR diagnostic developed by White *et al.* 2007 [21]. Frequencies in the pools were M-pool: 0.05/0.95 2La+/2La; and S-pool: 0.65/0.35 2La+/2La. Following determination of DNA concentrations using PicoGreen [22], pools containing equimolar amounts of DNA from each contributing sample were created and used for PCR.

### PCR and sequencing of pooled samples

Target loci were primarily selected to be coincident with the genes on the *An. gambiae *detox chip [13] with additional loci sequenced to cover the paracentric inversion polymorphisms on chromosomes 2L and 2R [23], which might aid future identification of inversion karyotypes from the linkage disequilibrium in these regions. Details of genes studied are given in Additional File 1. Primers were designed to generate amplicons of approximately 600 bp using Primer3 [24] and checked for unique binding to the Vectorbase-Ensembl AgamP3 genome sequence using BLAST. Our strategy was to amplify genic regions plus flanking regions approximately 5 kbp up- and down-stream in an attempt to capture variation potentially associated with nearby *cis *regulatory elements. Where genes were > 5 kbp in length, primers were designed to amplify regions approximately every 5 kbp. In total, 973 primer pairs were designed (including redesigned primer pairs to replace those which could not be optimised). Reactions were optimised to yield a single product, which was sequenced in both forward and reverse directions, using the amplicon-specific primers, by Macrogen (Macrogen Inc., Seoul, South Korea).

Sequence traces were aligned with CodonCode Aligner (CodonCode Corporation, Dedham, MA.) Traces, or portions thereof, with low Phred quality scores were automatically discarded. Nucleotide positions exhibiting polymorphism within or between template DNA pools, were identified with the aid of the mutation detection tool in CodonCode Aligner, and through manual inspection of all sequencing traces. We assigned a confidence score to each SNP identified: 1 = a SNP is identified with full confidence, being clearly apparent in both forward and reverse sequencing traces; 2 = a SNP is identified with confidence but with the caveat that only unidirectional sequence is available; 3 = a SNP is observed but with some cause for doubt, *e.g*. only unidirectional sequence with a relatively high background signal is available. Since sequencing was undertaken on PCR products of pooled DNAs, estimates of SNP frequency were based on a visual estimate of relative peak height in ambiguous positions.

All SNPs have been submitted to dbSNP (see Additional file 1 for *SS *numbers; *rs *numbers are scheduled to be available in build 129 or 130 of dbSNP).

### Validation of the pooling approach

Sequencing of the individual samples used to make up the pooled DNA was undertaken for thirteen amplicons (CYP6R1, COE18026, CYP4G17, COEjhe1F, COEB21998, CYP325A3, COEjhe4F, CYP325C1, CYP9K1-up, CYP6M4, CYP6M1_1 (2 overlapping amplicons), CYP6M1_2). Although some of these loci were chosen on the basis of biological interest, the only other criterion was that primers generated single, strong PCR amplicons. Thus, the loci should comprise a representative sample of our pooled sequences. Individual DNA samples were amplified using the identical primers to those used on pooled DNA templates. As before, individual sequences were aligned with CodonCode Aligner and mutations identified using the CodonCode Aligner mutation detection tool, with all calls checked by manual inspection. SNP frequency from individual DNAs was calculated and compared to the SNP frequency estimates obtained via sequencing of pooled DNA samples. In addition, to investigate how polymorphism in the mixed-template pools corresponded with polymorphism in natural populations, we also sequenced two pools (*N *= 5 each) of field collected specimens from a single southern Ghanaian population (Dodowa, Greater Accra region, all S form, 2La^+^/2La = 0.5/0.5) and two pools (*N *= 5 each) from Cameroon (Ngousso, Yaoundé, all M form, 2La^+^/2La = 1.0/0) using the same 13 primer pairs. Data from the two pools of *N *= 5 were combined for analysis.

### Data Analysis

Variability was calculated as the number of segregating sites (*S*) and the nucleotide diversity (*π*). Although allele frequencies were determined through pooling, *π *can be estimated [25] following Li [26]. We adjusted our segregating site frequency (*S*) and nucleotide diversity figures to account for the false positive (FPR) and false negative rates (FNR) identified through comparison of individual and pooled sequences (S = S_estimated_/1(1-FNR+FPR); *π *= *π*_estimated_/(1-FNR+FPR)). Variability was determined across the total dataset, and was also analysed following subdivision into five categories of loci: cytochrome p450 mono-oxygenases; glutathione-S-transferases; carboxy/cholinesterases; other detoxification loci; and other loci with no known detoxification function. Bootstrapped confidence limits of *S *were calculated using the Poptools add-in for Microsoft Excel [27]. In order to estimate whether SNPs were distributed evenly across the regions sequenced, gap distances (distance in base pairs between adjacent SNPs) were calculated for 5653 SNPs (following omission of sequences with only a single SNP and also the first and final SNP in each sequence). Each value (within a sequence) was then compared to the average gap distance for the whole sequence, yielding a count of gaps lower and higher than the average in each sequence; measures which are independent of SNP frequency. The null hypothesis of symmetry in the distribution of gaps around the average of each sequence was examined using a sign test in SPSS 14 (SPSS Inc.), performed across all sequences.

### Design of Illumina GoldenGate assay

All SNPs identified were submitted to the Illumina Assay Design Tool (ADT) to determine their suitability for genotyping with the GoldenGate assay. The ADT assesses whether an Illumina assay can be used to interrogate the SNP, checking for duplicated regions, SNPs in flanking sequence, and whether probe melting temperatures are within assay limits.

## Results

### Evaluation of the pooling approach

**Figure 1:**
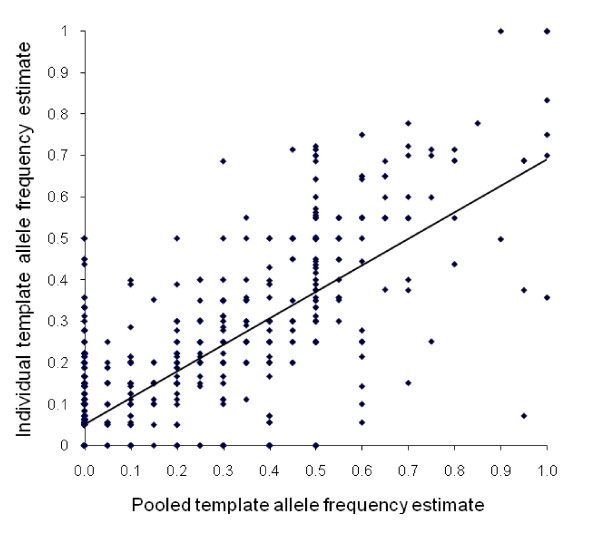
**Relationship between SNP frequencies estimated from sequencing of individual DNA templates and sequencing of pooled templates**. *R*^2 ^= 0.61, *P *< 0.001. False positives are arrayed along the x axis (y = 0) and false negatives are arrayed along the y axis (x = 0).

**Figure 2:**
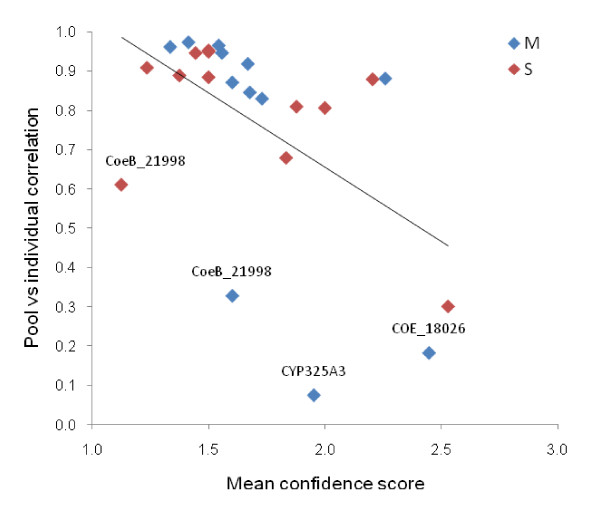
**Correlation between individual and pooled allele frequency estimates for M- and S-form pools plotted against confidence score (lower = more confident)**. *R*^2 ^= 0.27, *P *< 0.01. Points are labelled where sequence quality is poor and/or indel-affected.

**Figure 3:**
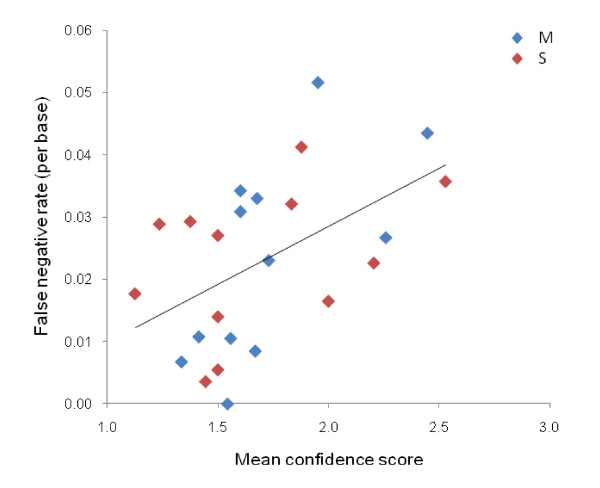
**False negative rates in pooled template SNP discovery for M- and S-form pools plotted against confidence score (lower = more confident – see text for definition)**. *R*^2 ^= 0.25, *P *< 0.05.

Overall, there was a good correlation between SNP frequencies estimated from pooled and individual data (Figure 1). The rate of false positives, *i.e*. SNPs identified through sequencing of pooled templates that were not confirmed by sequencing of individual samples, was low at 4.1%. Sequencing of pooled DNA generated a high rate of false negatives, with 30% of SNPs identified by individual template sequencing missed, but the median frequency of these SNPs was low (0.10; where x-axis = 0 in Figure 1). Across sequences the majority of correlations between individual and pooled allele frequency estimates were consistently high and well predicted by confidence scores (Figure 2). However, several relatively poor correlations are evident (labelled in Figure 2), which were either impacted by high indel frequencies (COEB21998, CYP325A3 M-pool – no indels present in S-pool) or were of marginal sequence quality (COE18026). Similarly, mean confidence scores showed significant predictive value for false negative rates (Figure 3). Perhaps most strikingly, false positive rates differed dramatically among confidence score classes with a 2.1% rate for confidence score 1 (37% of SNPs) and a 7.8% rate for confidence score 2 (47.5% of SNPs) but a 32.1% false positive rate for confidence score 3 (15.5% of SNPs). Clearly, therefore, SNPs assigned a confidence score of 3 should be avoided as target markers unless additional evidence of polymorphism is available.

### Relationship between polymorphism in M and S pools and single population samples

Allele frequencies estimated from the diverse S-form pool correlated well with those in pools of samples from a single population collection of S-form samples from Ghana (median Pearson correlation across sequenced amplicons *r *= 0.80) and moderately well with those in a pool of M-form samples collected from a single population in Cameroon (median *r *= 0.51). Allele frequencies in the diverse M-form pool were moderately correlated with those in the Cameroon single population pool (*r *= 0.42), and only weakly related to those in the Ghanaian single population pool (*r *= 0.21). Correlations between the allele frequencies obtained from sequencing individuals comprising the M and S-pools and the Cameroon and Ghanaian pools were similar to those obtained when comparing pooled estimates. Thus SNPs identified in the diverse S-form pool could be of greater general utility than those in the diverse M-form pool, though those present in both M- and S-form pools are likely to be of most widespread value.

### Properties and frequency of segregating sites

In total, sequencing was undertaken successfully on 660 loci (see supplementary material), comprising 323,114 bp. Other PCR reactions failed to optimise, gave unusable sequence or were affected by multiple indel events which prevented analysis. Sequencing of geographically diverse pools of M (*N *= 10) and S (*N *= 10) individuals revealed a total of 7062 polymorphic features. Sixty-seven (0.95%) were indels, of which we could not determine the exact position for 36. Additional indels were inferred from rapid reductions in quality of sequencing traces, but neither the causative polymorphism nor its exact position could be determined. These are not included in the polymorphic feature count and thus we will have underestimated the true indel frequency. The remaining 6995 polymorphic features were SNPs. 702 of the 7026 features (10%) already have dbSNP numbers from sequencing of the PEST strain, whereas 6324 are novel. Sixty-seven triallelic and three tetrallelic SNPs were identified directly from sequencing traces. An additional 15 SNPs were inferred to be triallelic through discrepancies between the nucleotide variation we identified and that identified at the same SNP position via sequencing of the PEST strain. Thus, we estimate that approximately 1% of all SNPs are multiallelic.

**Figure 4:**
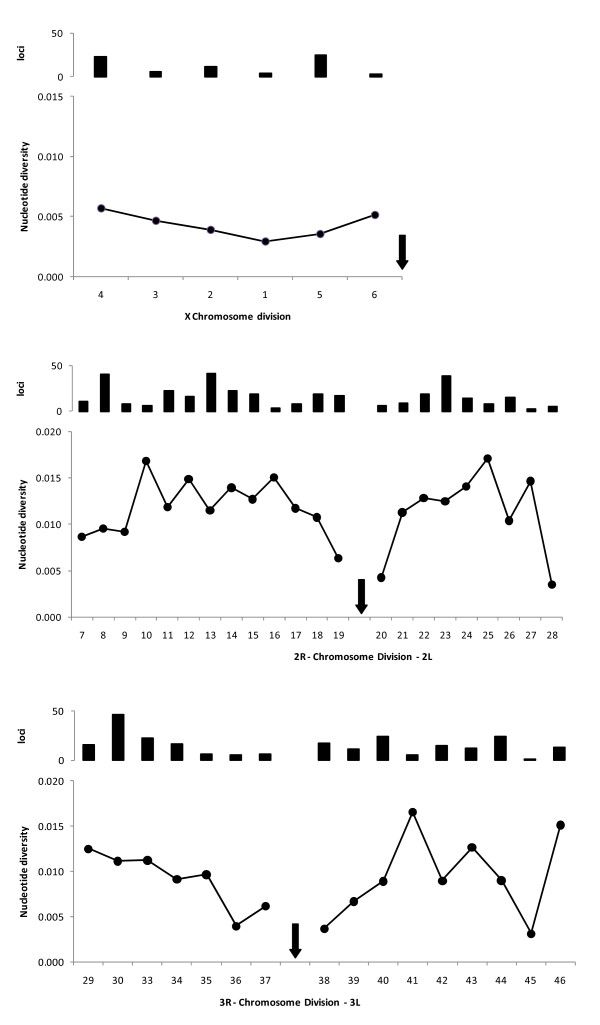
**Nucleotide diversity plotted against chromosome division in *An. gambiae *(average of M- and S-form pools); with bars showing the number of loci sequenced in each chromosome division**. Approximate positions of centromeres are marked by arrows. Note the upper plot is scaled to reflect the lower effective population size (3/4) of the X chromosome.

**Figure 5:**
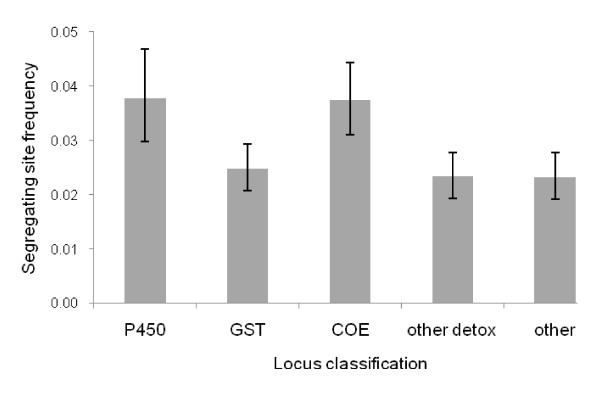
**Frequency of segregating sites (S) in *A. gambiae *by gene class – cytochrome p450s (p450), glutathione-S-transferases (GST), carboxy/cholinesterases (COE), other detoxification loci (other detox) and control non-detoxification related loci (other)**. Error bars are 2.5% and 97.5% confidence limits, generated by bootstrapping.

A polymorphic feature was found approximately every 34 bp (after correction), although lower frequencies of segregating sites were observed on the X chromosome and around centromeres (Figure 4). Nucleotide diversity was similar in the M (*π *= 0.0079) and S pools (*π *= 0.0082) but there was significant variation among gene classes (ANOVA *F*_4,655 _= 13.2, *P *<< 0.001) with the cytochrome P450s and carboxylesterases much more polymorphic than other classes (Figure 5). Of the 7062 polymorphisms, 191 (or 2.7%) were found to differentiate the M and S- form pools. Whilst it would be unwise to regard these as fixed M- vs S-form differences because of the small sample sizes involved, it is interesting to note that many of these SNPs are located within the 'islands of speciation' [28] on chromosomes 2L, X and 2R.

### SNP distribution

**Figure 6:**
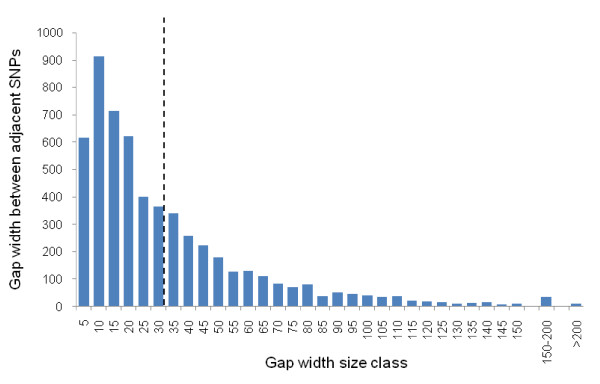
**Distribution of gap distances between adjacent SNPs**. The dashed line indicates the overall mean gap width.

By comparing the distribution of gap lengths (distances between features) to the average for each sequence we found that 60% of gap sizes fell below the average, with little variation among the locus classes (p450s, GSTs, COEs, other detoxification, control loci: range 59%–61%). The distribution of gap widths was significantly less symmetric about the averages than expected by chance (Sign test *z *= -12.854; *P *<< 0.001), indicating significant clustering of SNPs, with many exhibiting very small gap distances (Figure 6). Thus, SNPs are clustered but not in a locus-type specific way, suggesting that, in contrast to SNP frequency, SNP clustering is a general feature of the genome, rather than being confined to certain gene families.

### Illumina GoldenGate Assay design

**Figure 7:**
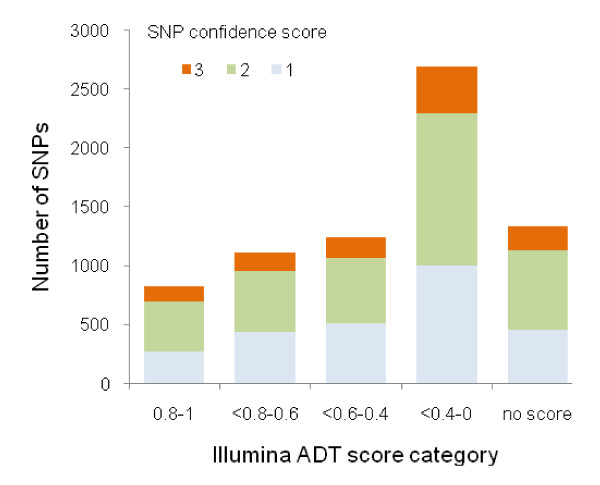
**Classification of SNPs according to Illumina's assay design tool (ADT) algorithm, which predicts suitability in the Illumina Goldengate assay, and SNP confidence scores (lower = more confident)**. SNPs with higher ADT scores are predicted to have a better chance of success.

To determine if the SNPs could be used for association mapping studies on the Illumina Goldengate platform we used Illumina's assay design tool (ADT) algorithm (Additional file 1). Results are summarised in Figure 7. Only 4% of all SNPs met the highest quality criteria for design recommended by Illumina: well validated (represented by our confidence score 1) and an ADT score of ≥ 0.8. However, 23% of SNPs were considered suitable for assay design with ADT scores ≥ 0.6 and confidence scores of 1 or 2, since false positive rates for confidence score 2 are reasonably low (see above). ADT scores between 0.4 and 0.6 were less favoured but such SNPs may be used for design (SNPs with an ADT score < 0.4 are not recommended): 38% of SNPs with a confidence score 1 or 2 had an ADT score of ≥ 0.4. Illumina recommends that SNPs to be targeted in the Goldengate assay should have a minimum of 50 bases of flanking sequence free of polymorphism on each side: the high and clustered nature of polymorphisms meant that only 5% of our SNPs fulfilled this criterion. Nevertheless, it is interesting to note that, some flexibility in placement of one of the probes (the so-called locus-specific oligonucleotide) in the assay design clearly permits many more acceptable assays to be designed than predicted by this strict 50 bp per flank criterion.

## Discussion

The development and application of high-throughput genotyping methodologies for the malaria mosquito *Anopheles gambiae *depends upon the identification of SNP markers. We have resequenced approximately 0.12% of the *An. gambiae *genome in geographically diverse pools of *An. gambiae *M- and S-forms, identifying 6,995 SNPs and 31 indels that could be mapped, and 36 indels that could not be precisely mapped (additional indels were inferred but could not be precisely identified or positioned due to their effect on sequence quality). Of the SNPs we identified, only 10% had been identified previously from sequencing of the PEST strain genome. This suggests that the sequencing of this strain has dramatically underestimated the true SNP frequency in *An. gambiae*. Similarly, Morlais *et al*., in sequencing of 3 lab strains (Yaoundé, L35, 4arr), found 324 SNPs in 26 loci (total 17 kbp) [11]. Only 39% of these SNPs had been predicted by Ensembl (although Ensembl records an additional 42 not observed by Morlais *et al*. [11])

By sequencing the gDNA of pooled individuals we substantially reduced the cost of the resequencing programme. Through comparison of allele frequencies estimated from pooled DNAs with those obtained from sequencing of individual templates it is apparent that pooling of template DNAs yields relatively accurate allele frequency estimates and a very low rate of false positives. Many low frequency SNPs that were identified through sequencing of individual DNA samples were missed in the sequencing of pooled templates. However, since low frequency SNPs perform poorly in detection of linkage disequilibrium [29] this is unlikely to be problematical when identifying SNPs suitable for use in association mapping studies. Though essentially qualitative, our SNP confidence scores proved valuable predictors of false positive rates, and should be considered when choosing from the SNPs we have identified, noting that SNPs with category 3 confidence scores are much less likely to be truly polymorphic than those with confidence scores 1 and 2. In summary, pooling of gDNA templates provided a useful technique in permitting analysis of polymorphism at a large number of genes in a total of 20 individuals (as two pools of 10 each), at one tenth of the cost of individual sequencing. If cost-reduction is not a major consideration and/or if detection of low frequency polymorphisms is a primary concern, sequencing of individual templates or the use of a next generation technology, such as 454 pyrosequencing (454 Life Sciences), with pooled PCR products would be a preferred approach.

Nucleotide diversity estimates in our study are comparable to those obtained in other studies of *An. gambiae *[10,11,30,31] or other mosquitoes [32] (Table 1), particularly those employing similar sample sizes [10,11]. Indeed, the only study recording much lower diversity [30] involves either extremely low sample sizes or loci in a known area of low recombination (Table 1). It is interesting to note that we observed the same pattern as Cohuet et al. [11] with respect to X-chromosome diversity: even allowing for smaller effective population size (3/4) of the X chromosome than the autosomes, nucleotide diversity is low. However, we did not observe the dramatically lower diversity in X chromosome divisions 5 and 6, than divisions 1–4 reported by Stump et al. [30]. We suspect that the greater degree of mixing of distinct populations in our study might reconcile these findings, since slowly recombining regions will tend toward loss of diversity within, and increased differentiation among, populations. Mixing of populations will thus have a proportionately greater impact in such a genomic area since differentiation will inflate measures of diversity.

**Table 1:** Estimates of nucleotide diversity in mosquitoes (), obtained from different source populations, numbers of loci sequenced (*N *loci) and sample sizes (*N*).

Species	Source of sequenced samples	*N *loci	*N*		Study
*An. gambiae ss*	mixed wild population (M)	8	20^3^	0.0208	[30]
	mixed wild population (M)	14^2^	20^3^	0.0043	[30]
	single wild population (M)	109	8	0.0076	[10]
	mixed wild populations (M)	660	10^4^	0.0079	Present study
	3 lab strains (M)	35	7–9	0.0091	[11]
	mixed wild populations (S)	8	22^3^	0.0191	[30]
	mixed wild populations (S)	14^2^	22^3^	0.0043	[30]
	mixed wild population (S)	109	9	0.0092	[10]
	mixed wild populations (S)	660	10^4^	0.0082	Present study
*An. arabiensis*	single wild population	22	2^3^	0.0040	[30]
	lab population^1^	109	8	0.0064	[10]
*An. funestus*	single wild population	50	21	0.0079	[31]
*Ae. aegypti*	3 lab strains	25	n/a	0.0122	[32]

^1^recently colonised from field without dramatic bottleneck [10]; ^2^centromeric X-chromosome loci; ^3^average *N *across loci; ^4^single pool of DNA:  corrected for elevated Type II error rate.

Polymorphism estimates based upon nucleotide diversity are less informative than the frequency of segregating sites for the design of high-throughput assays where variable bases close to the SNP of interest can affect assay design and therefore should be avoided. On average we find a segregating site every 34 bp, a figure which compares favourably with previous estimates from mosquitoes. Apart from the aforementioned exceptional figures associated with centromeres or a small sample, the range of estimates for segregating site frequency for the studies cited in Table 1 are 1 SNP per 29 to 1 SNP per 48 bases. The problems for assay design resulting from this high SNP frequency will frequently be exacerbated because SNPs show a clustered distribution. Unrecognised non-target SNPs in probe-binding sites can appear as null alleles in Illumina analyses [33,34]. Whilst their effects on the use of Affymetrix Genechips for genotyping are unknown, non-target SNPs are detrimental to gene expression profiling on this platform [35,36]; it is reasonable to assume they may also negatively affect genotyping accuracy. In addition to the impact of high SNP density, the effect of multiallelic SNPs must also be recognised for probe design. Multiallelic SNPs will also pose difficulties for genotyping with multiplex genotyping platforms as null alleles will be scored. Although null alleles can be recognised with some platforms, and controlled for [33,34], they could be problematical where not anticipated.

GoldenGate assays have, to date, been successfully applied to a variety of species, including humans, honey bee [37], cattle [38], spruce [39], soybean [40] and barley [41]. Conversion rates of assays have been consistently high for these species, indicating that secondary polymorphisms or unrecognised multiallelic SNPs have not had a major impact on study success. However, all of these species either exhibit low polymorphism or studies were undertaken on inbred lines. For example, in the human genome, SNPs occur on average at 250 bp intervals (Ensembl 50 human genome statistics). Therefore, the high SNP frequency in *Anopheles*, and the coincident effect on Goldengate assay design, is a far more significant problem than for previous studies. Indeed, according to Illumina's assay design tool, the majority of SNPs were unsuitable for Goldengate assay probe design.

The *Anopheles*/*Plasmodium *Affymetrix Genechip, which was designed for gene expression studies, rather than as a genotyping tool, has been used to study the degree of differentiation between the M and S forms [28]. Since the probe length for this assay is shorter (25 bp) than in the Illumina GoldenGate assay, the high SNP frequency may be less problematical. However, since the array was not designed specifically for genotyping it is difficult to assess the inherent difficulties posed by the high diversity and clustering in *Anopheles *for this assay. Although quantitative extrapolation of our array design experience with Illumina to other platforms is difficult, it seems clear that for *Anopheles*, and probably other mosquitoes or species with high rates of genomic diversity, high throughput SNP-typing will be negatively impacted, through loss of SNPs at the design stage and/or loss of data due to null alleles at the analysis stage. Whilst somewhat speculative, it also seems likely that confident assembly of short-read fragments into contigs or onto the template of an existing genome assembly in massively parallel sequencing runs [42] will be rendered difficult if multiple SNPs are present in many fragments. Hopefully, a more comprehensive database of segregating sites in *An. gambiae *might ameliorate this problem.

In the present dataset, SNP frequencies varied both physically and according to their location within or near gene classes. As reported elsewhere [30] and predicted by lowered recombination rates within the regions, diversity was lower toward the centromeres of autosomes and on the X chromosome. Diversity was significantly elevated in loci of the cytochrome p450 mono-oxygenase and carboxy/cholinesterase (COE) families than in the glutathione-S-transferases and control loci, with a segregating site every 26 bp in the p450s and COEs compared with every 34 bp overall. This higher SNP frequency is likely to exacerbate the problems for assay design in these gene families, especially given the significant SNP clustering in this genome. High rates of variability in human p450s have been reported [43] but higher rates of polymorphism in mosquito p450s or COEs have not been previously identified.

A higher rate of insertion of transposable elements in xenobiotic-metabolising p450s of *Drosophila *(in contrast to those p450s involved in ecdysone biosynthesis and developmental regulation) result in high rates of mutability of p450s [44] indicating that the function of such p450s is more tolerant of polymorphism. Also in *Drosophila*, enzymes involved in xenobiotic metabolism exhibit a higher nonsynonymous: synonymous (*dN*/*dS*) ratio than the average over the dataset (ω = 0.05 compared with ω = 0.045 overall, *P *= 0.011 [45]). The higher levels of *dN*/*dS *for xenobiotic enzymes may indicate that the higher polymorphism levels seen in p450s and COEs reflects less stringent selection at these loci than others, perhaps because of flexibility in function among closely-related gene family members.

The high diversity in *An. gambiae *is likely related to large effective population size (*Ne*). Nucleotide diversity is a product of mutation rate and *Ne *and the highest recorded levels of polymorphism, for the urochordate *Ciona savignyi*, are thought to be due to its high *Ne *[46]. The estimates of *Ne *available for *An. gambiae *suggest levels of *Ne *equal to a few thousand [47,48]. However, *Ne *is notoriously difficult to estimate accurately, particularly for species exhibiting often limited genetic population structure over wide geographic scales, such as *An. gambiae*. Improved *Ne *estimates would help elucidate the role of *Ne *in explaining the high nucleotide diversity that we, and other authors, have observed.

In *Drosophila *spp. recombination rates are positively correlated with nucleotide diversity [49-51], especially at a fine-scale [51], although the relative roles of selection and mutation generated by recombination in underpinning the pattern are controversial [49-51]. In *An. gambiae*, the first major study to estimate recombination rate indicated a small recombination map length of 215 cM over the 278 Mb genome, or 0.78 cM/Mb [1]. This is lower than typical average figures of 1–4 cM/Mb for most organisms and far less than the 19 cM/Mb recorded in the honey bee [52]. Thus, broad-scale recombination estimates in *An. gambiae *do not support a relationship between diversity and recombination rate. However, more recently, a survey of recombination rate along the X-chromosome, recorded an overall average recombination rate of 1 cM/Mb, but with dramatic variation in local rates between 0.2 and 7 cM/Mb [53] dependent on chromosome position. Thus a link between sporadically high recombination rates – perhaps involving recombination hotspots – and high, clustered diversity could apply in *An. gambiae*. Fine-scale estimates of recombination rate are now required to permit investigation of how the interplay between recombination and selection determines diversity.

## Conclusion

By sequencing pooled template DNA, we have identified nearly 7000 SNPs in *Anopheles gambiae*, primarily in or around detoxification-related genes. SNP frequencies varied among gene families, being particularly high in members of the P450 monooxygenase and carboxyl/cholinesterase enzyme superfamilies. The SNPs identified represent a valuable resource for mapping studies, but a high SNP frequency and clustered distribution in *An. gambiae*, which may be general features of mosquito genomes, present a significant challenge for the design of genotyping arrays.

## Authors' contributions

CSW designed PCR assays, performed sequence alignments, analysed the data and wrote the manuscript. DW designed PCR assays, performed sequence alignments, analysed the data and co-wrote the manuscript. KS carried out molecular laboratory work. MJD participated in the design and coordination of the study and contributed to the manuscript. All authors read and approved the manuscript.

## Supplementary Material

Additional file 1 **Loci studied and snps identified** Click here for file
